# 3-Dimensional characterization of cortical bone microdamage following placement of orthodontic microimplants using Optical Coherence Tomography

**DOI:** 10.1038/s41598-019-39670-9

**Published:** 2019-03-01

**Authors:** Hemanth Tumkur Lakshmikantha, Naresh Kumar Ravichandran, Mansik Jeon, Jeehyun Kim, Hyo-Sang Park

**Affiliations:** 1 Deptartment of Orthodontics, School of Dentistry, Kyungpook National University, Daegu, 41940 South Korea; 2 School of Electronics Engineering, College of IT Engineering, Kyungpook National University, Daegu, 41566 South Korea

## Abstract

Microimplants are being used extensively in clinical practice to achieve absolute anchorage. Success of microimplant mainly depend on its primary stability onto the cortical bone surface and the associated Microdamage of the cortical bone during insertion procedure leads to many a microimplants to fail and dislodge from the cortical bone leading to its failure. Even though, previous studies showed occurrence of microdamage in the cortical bone, they were mainly 2-dimension studies or studies that were invasive to the host. In the present study, we used a non-invasive, non-ionizing imaging technique- Optical Coherence Tomography (OCT), to image and analyze the presence of microdamage along the cortical bone surrounding the microimplant. We inserted 80 microimplants in two different methods (drill and drill free method) and in two different angulations onto the cortical bone surface. Images were obtained in both 2D and 3D imaging modes. In the images, microdamage in form of microcracks on the cortical bone surface around the bone-microimplant interface and micro-elevations of the cortical bone in angulated microimplant insertions and the presence of bone debris due to screwing motion of the microimplant on insertion can be appreciated visually and quantitatively through the depth intensity profile analysis of the images.

## Introduction

With the advent of microimplants in orthodontics, we are able to achieve a reliable and sustainable three-dimension anchorage^1^. For a successful outcome of an orthodontic treatment using microimplants, it is vital to achieve an adequate primary stability, followed by a sustainable period of secondary stabilization of the microimplant. Cortical bone anatomy is one of the most important factor affecting primary stability. In general, microimplants are inserted on the cortical bone with an ideal angulation for required orthodontic movement. However, in some cases, the anatomy and morphology of the alveolar bone or the desired direction for the movement of the need might dictate the angulation of the microimplant. The insertion technique, such as the insertion angle and insertion method play a vital role in attaining primary stability. Park *et al*.^2^, introduced angular placement of microimplants to minimize root contact and gain access to more bone surface at the apical region and thereby increasing primary stability. Predrilling reduces insertion torque, especially as the depth of predrilling increases and in more dense bones, and enhances primary stability^3–5^. Mechanical interlocking of the microimplant with the surrounding cortical bone, provides primary stability to the microimplant, additionally also caters significant microdamage onto the structure of the cortical bone. Previously, numerous studies have been conducted to study the occurring microdamage using different modalities. But these studies were either invasive to the bone or were biomechanical analysis models simulating the stress patterns on the bone. Moreover, these studies only studied the occurring microdamage, which is a 3-dimensional entity in a 2-dimensional manner.

3-Dimensional imaging has greatly evolved and found a lot many applications in orthodontics and in dentistry in general. In 3D medical imaging process, a set of data collected via a diagnostic imaging equipment is processed by a computer and the output is projected onto a 2D monitor to give an illusion of depth. Depth perception causes the image to appear in 3D^6^, thus overcoming the limitations of 2D static imaging techniques. Therefore, incorporation of 3D imaging and analysis of the 3D becomes vital in the future of orthodontic research. Numerous diagnostic methods have been developed to project the craniofacial structure in all three dimensions, but many were abandoned due to their various drawbacks. Currently in use, the most popular methods are Computerized tomography (CT), Cone Beam Computerized Tomography (CBCT), Micro Computerized Tomography (MICRO-CT), 3D laser scanning, Tuned‐Aperture Computed Tomography (TACT), and Magnetic Resonance Imaging (MRI). Each of these techniques have its own drawbacks, mainly being invasive to the subject and production of images with subpar quality. Therefore, we chose to utilize a non-invasive and non-destructive imaging system with a high resolution and high contrast, capable of producing 3-dimensional images, such as the optical coherence system to visualize and analyze the microdamage occurring in the cortical bone due to microimplant placement.

Optical coherence tomography (OCT) is an imaging system, which provides volumetric and cross-sectional images to disclose the internal structure of biological tissues non-invasively and non-destructively^7^. OCT is based on the more than 100-year-old principle of Michelson interferometry. The technique was first introduced by Fercher *et al*.^8^, and Huang *et al*.^9^, for investigation of the human eye. Application of OCT as a non-invasive imaging tool has been diversely used in agronomy^10–12^, entomology^13,14^, industrial inspection^15^, and ophthalmology^16–18^ has been studied for more than two decades. In the field of orthodontics, it has proven to be of value for visualization of changes in the enamel surface after routine orthodontic de-bonding and various demineralization procedures^19–22^. The ability of the system to provide a three-dimensional (3D) images and two-dimensional (2D) images with high sub-micron resolution is of note. Possibility of real time imaging, makes it an exciting new system in the field of orthodontic research.

The purpose of this study was to characterize the microdamage occurring in the cortical bone surface following placement of an orthodontic microimplants with different placement modalities and at different angulations to the cortical bone surface and to present data as a 3-dimensional qualitative and quantitative manner. As a means to an end, we initially developed a mathematical model (FEA), on the basis of which, we analyzed the images after the experiment (OCT) and confirmed our result with micro-computerized tomography, which is widely regarded as a gold standard when it comes to hard tissue imaging.

## Results

**Figure 1:**
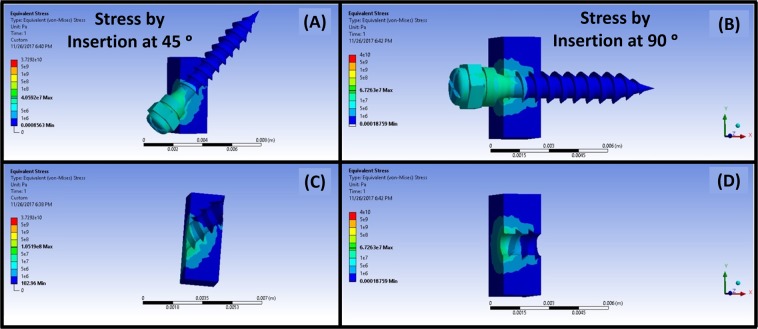
Representative image of the FEA model, showing stress patterns along the bone-microimplant interface. (**A**,**C**) Shows FEA simulation of stress pattern for microimplant insertion at 45°. Likewise, (**B**,**D**) shows the FEA simulation of stress pattern for microimplant insertion at an angle of 90° to the cortical bone surface.

Figure 1, represents the finite element model created to simulate the stress patterns around the microimplant post insertion onto the cortical bone surface. The stress values are shown as resultant von misses stress. Based on the simulation we can concur that, the majority of stress accumulation is seen at around the subsurface of the microimplant and around the head of the microimplant. When the microimplant is placed at an angle, there is accumulation at the lesser angle created by the microimplant and the bone surface. Microimplant placed at 90° created lesser stresses as when compared to at an angle of 45°.

### OCT imaging for bone damage evaluation upon microimplant placement

All bone samples were separated into two main groups, in the first group, the microimplant were placed directly on to the bone surface and in the other, and a pre-insertion hole was drilled before microimplant insertion. These two groups were further sub-divided based on the angle on to which the microimplants were inserted, i.e., 45° and 90° to the cortical bone surface.

**Figure 2:**
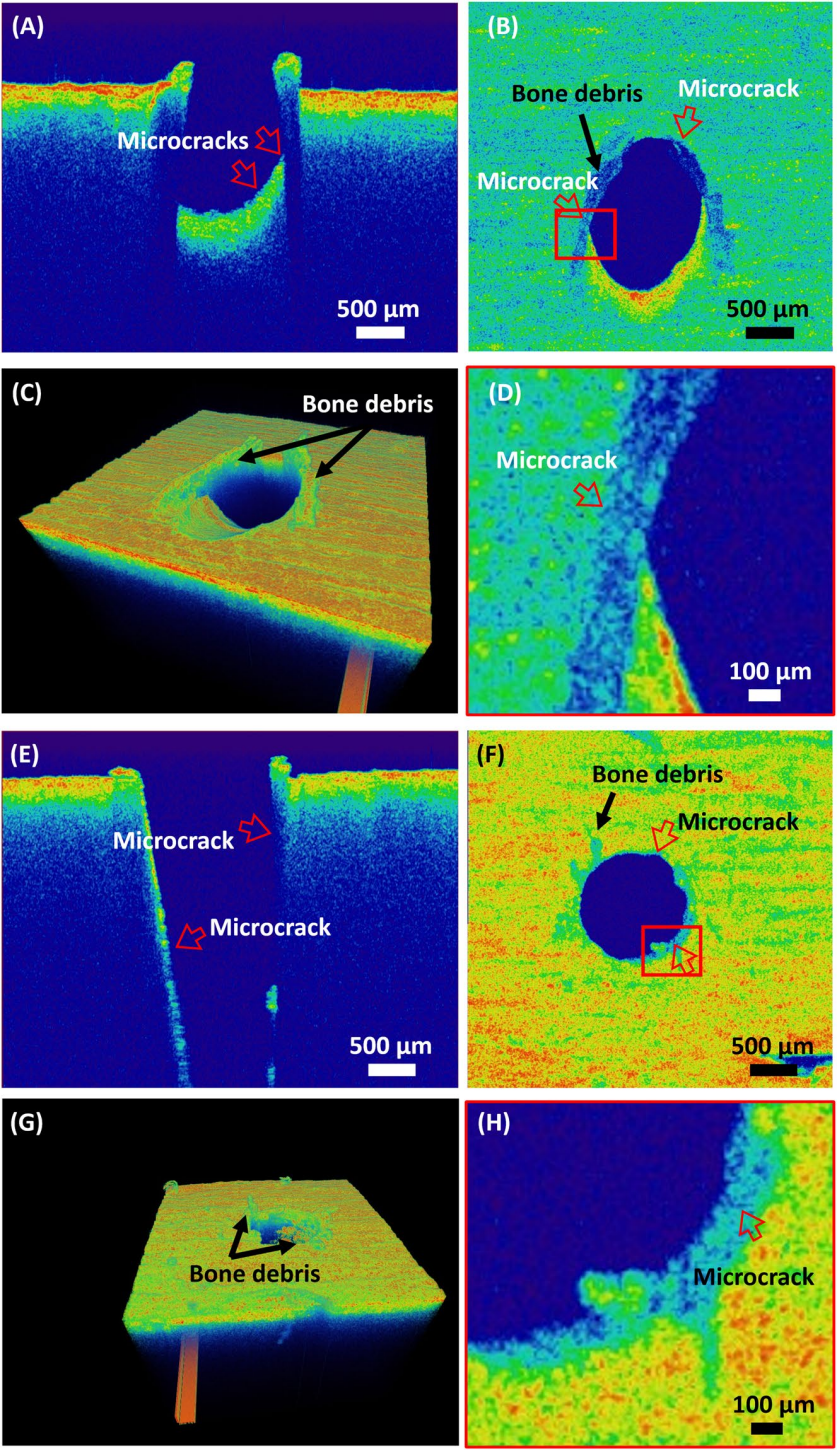
2D and 3D OCT images of bone samples after pre-insertion drilling of the cortical bone surface. (**A**,**B**,**E**,**F**) Are OCT images of a cortical bone surface which was pre-insertion drilled at 45°. Similarly, (**C**,**D**,**G**,**H**) are OCT images of a cortical bone sample which was pre-insertion drilled at an angle of 90°. Red arrows indicate cracks. (**F**,**H**) Are enlarged images of red box region shown in (**B**,**D**) respectively.

Figure 2 shows the 2D and the 3D volumetric OCT images of representative bone samples which were taken after the pre-insertion hole drilling procedure. In Fig. 2(A–D) are OCT images of the bone sample in which a pre-insertion hole at an angle of 45° to bone surface was placed. Image (A) is the 2D cross-sectional OCT image, whereas (B) is the *en face* image of a 3D volumetric image at 100 µm depth. (C) is the 3D volumetric image that was reconstructed from the obtained successive 2D images in lateral direction within the scan region. (D) is the enlarged image of the red box region shown in Fig. 2(B). Red arrows heads in the images indicate the cracks on the bone, that occurred due to the drilling procedure with a bur. Likewise, in Fig. 2(E–H) are OCT images of the bone sample in which a pre-insertion hole at an angle of 90° to bone surface was placed. Correspondingly the 2 D cross-sectional image is shown in Fig. 2(E,F) is the *en face* image of a 3D volumetric image at 100 µm depth from the top surface of bone. (G) and (H) are the 3D volumetric image and enlarged image of the red box region shown at Fig. 2(D). We can observe that the cracks occur at both pre-insertion drilling with angulations of 45° and 90°, even though the overall bone damage is notable higher at 45°.

**Figure 3:**
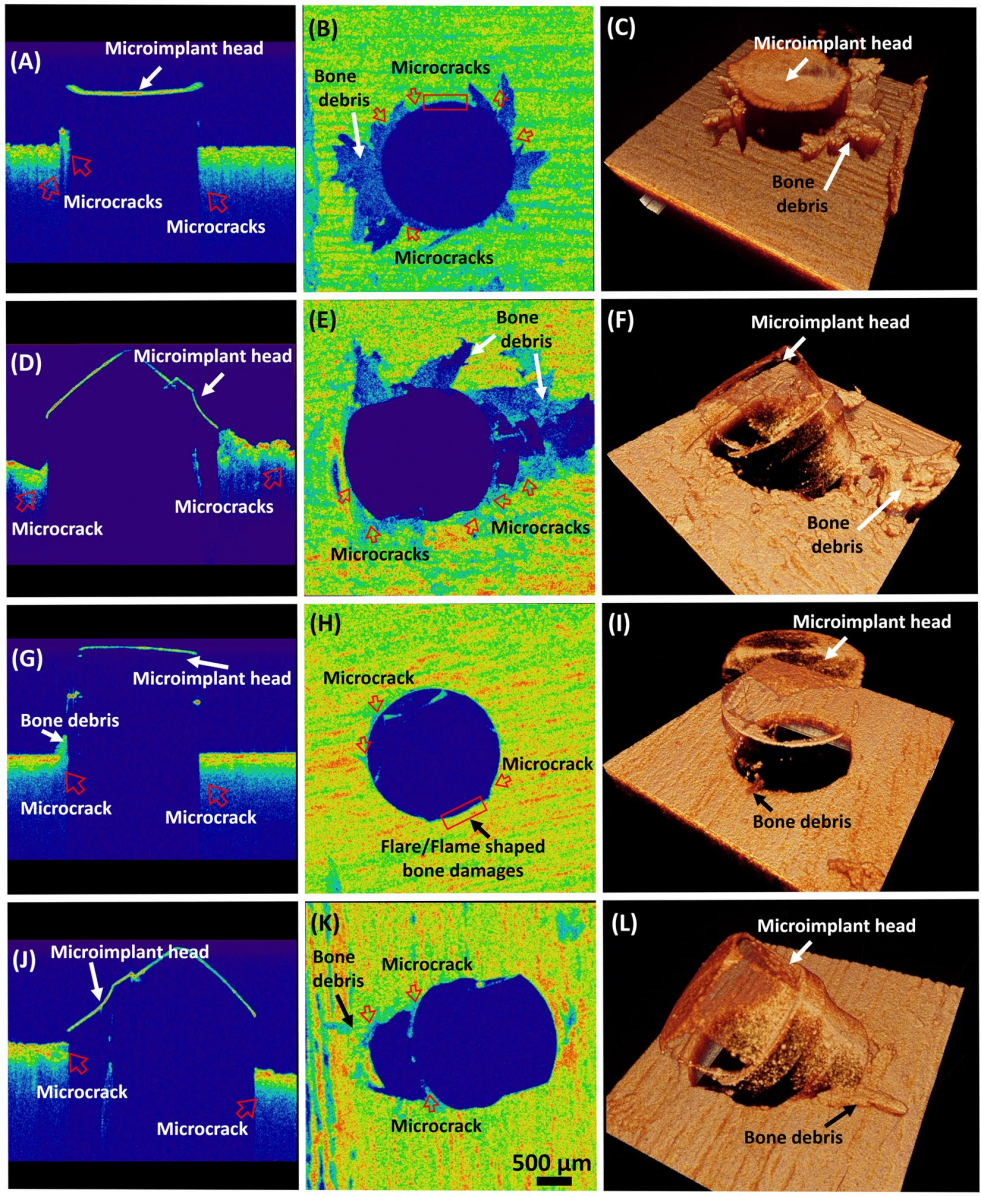
OCT image showing crack occurrence after microimplant insertion at 45° and 90°, with and without pre-insertion drilling. (**A**–**C**) and (**D**–**F**) are OCT images of microimplant inserted at 90°, and 45° respectively. (**G**–**I**) and (**J**–**L**) are OCT images of microimplant inserted at 90°, and 45° respectively after pre-insertion drilling was performed. Red arrows indicate cracks. And red rectangular boxes indicate the flares/flame shaped bone damages due to microimplant placement.

Figure 3 shows the assessment of bone damage at post microimplant insertion at the cortical bone surface. Images (A) to (C), are the respective 2D, *en face* and 3D OCT images of a representative bone sample of the sample group which were mounted with microimplants at 90° without pre-insertion drilling procedure. Likewise, (D) to (F), are the 2D, *en face* and the 3D OCT images of a bone sample that was placed with a microimplant at 45° respectively without pre-insertion drilling. Also, (G) to (I), and (J) to (L), are the 2D, *en face* and 3D OCT images of a respective representative bone sample of each sample groups in which a pre-insertion drill was performed before the microimplant was inserted at 90°, and 45° respectively. All the *en face* images were taken at 100 µm depth below the bone surface. The red arrows indicate cracks which occurred after implant placement, and red rectangular boxes indicate the flares/flame shaped bone damages which resulted by the microimplant placement. It is to be noted that the bone microdamage occurrence following microimplant insertion was comparatively higher in the group where no pre-insertion drilling was employed. Furthermore, it is also to be illustrated that during the OCT imaging of 45° implant placement, the bone structures which are directly beneath the implant head could not be scanned as this is due to the highly reflective property of infra-red light by the microimplant material. The microcrack occurrence was larger in 45° group, and the bone debris occurrence was more profound in the no-drilling microimplant group.

**Figure 4:**
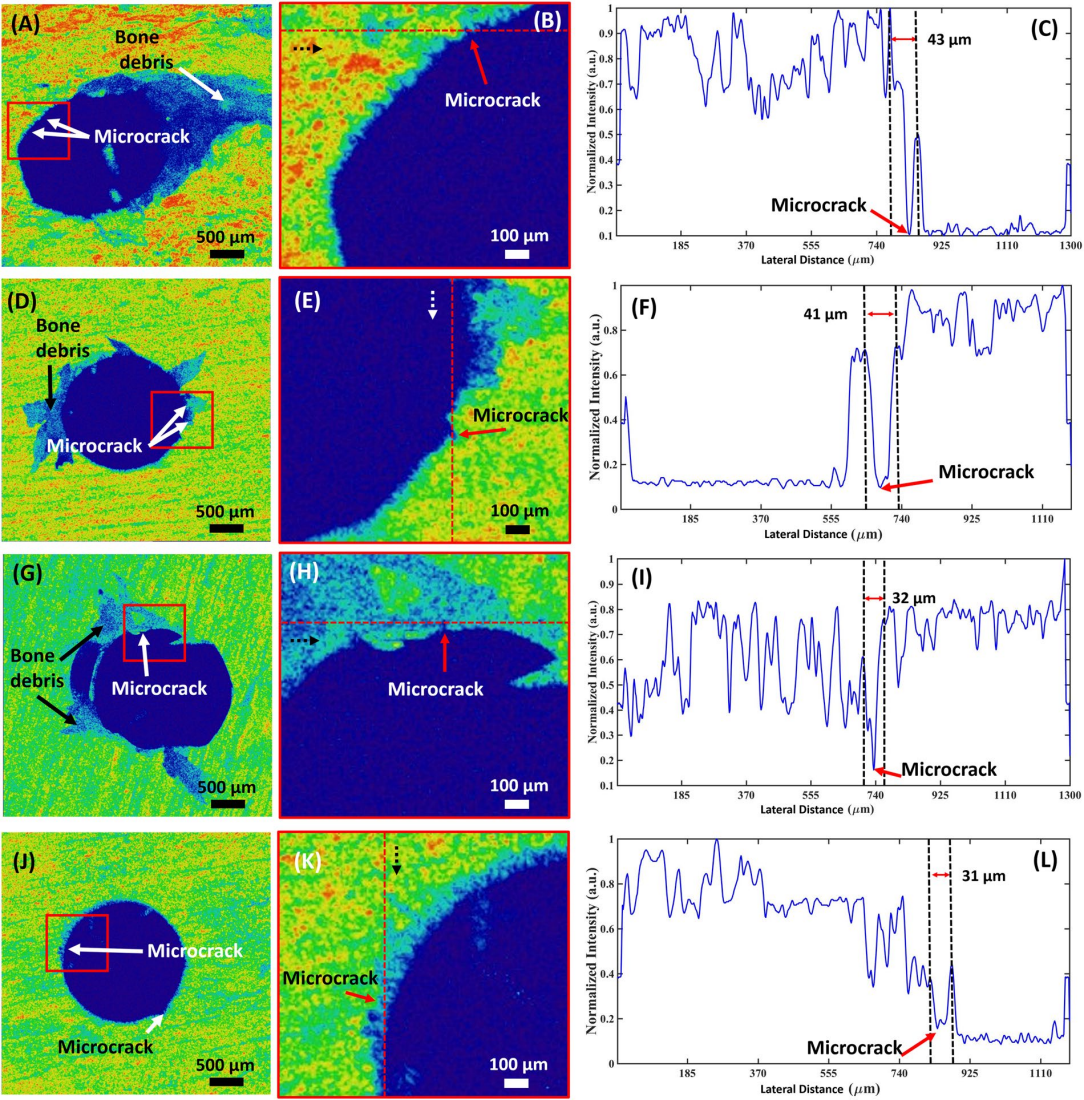
OCT images along with its depth intensity profile analysis for microimplant inserted at 45° and 90°, with and without pre-insertion drilling. (**A**–**C**) and (**D**–**F**) are *en face* images and depth intensity profile plots of cortical bone surface with microimplant inserted at 45°, and 90° respectively. Similarly, (**G**–**I**) and (**J**–**L**) are *en face* images and depth intensity profile plots of cortical bone surface with microimplant inserted at 45°, and 90° respectively after pre-insertion drilling was performed. Red arrows indicate cracks. Dashed arrows indicate direction of depth intensity profiles.

Figure 4 presents the representative 2D OCT images and their respective depth profile intensity plots for the four experimental groups. Images and plots in the Fig. 4(A–F) are the *en face* image (left) taken from a 3D volumetric OCT image, enlarged image (middle) as shown in rectangular red box region in the OCT image, followed by depth profile intensity plots (right) of the point indicated by staggered red line in the enlarged image, for the microimplant placed at 45°, and 90° respectively. Images and the plots shown in (A) to (F) belong to the no-drilling group. Likewise, Fig. 4(G–L) are the *en face* image (left) taken from a 3D volumetric OCT image, enlarged image (middle) as shown in rectangular red box region in the OCT image, followed by depth profile intensity plots (right) of the point indicated by staggered red line in the enlarged image, for the microimplant inserted at 45°, and 90° angle, after a pre-insertion drill was performed. The *en face* images were obtained at a depth of 100 µm from the surface of the 3D volumetric image.

In the depth intensity profile analysis, the sudden or abrupt intensity drop in the depth intensity profile plots indicates a microcrack. In all the intensity plots shown in Fig. 4, these sudden intensity drops are indicated by a red arrow, and the corresponding crack is shown in the enlarged *en face* OCT images. To measure the width of the microcrack, the distance between the successive highest intensity peak before and after the intensity drop is measured. The staggered arrows in the enlarged *en face* images (B), (E), (H), and (K) shows start to end direction of the intensity plots.

The representative table, Table 1 depicting the average crack thickness measured in en face OCT images of samples at 100 µm below the bone surface for the four experimental groups. Along with the average crack thickness/width found in each group, its respective standard deviation values are given. Also, maximum micro-elevation occurred due to microimplant insertion was calculated from cross-sectional image which was taken from the Volumetric OCT images. Data processing included the entry of all results into Statistical Package for the Social Sciences (SPSS) (Version 21.0). Non-parametric statistics were used, where required, since data were ordinal and data were unequal. A Mann-Whitney U Test was performed to compare between groups. The results were considered statistically significant if the p-value was < 0.05. The total number of prominent microcracks were counted in each sample in the en face OCT images at the 100 µm below the bone surface is illustrated in the table. Also, it is to be noted that the total number of measured microcracks in 45° groups were only taken from one side of the microimplant (towards the mounting direction). This is due to the fact that bone structures which are directly beneath the implant head could not be scanned as this is due to the highly reflective property of infra-red light by the microimplant material. Since, numerous minute microcracks could be seen in the OCT images, in order to avoid any false positive results, we included only the prominent microcracks that could be visibly seen to progress/propagate to at least 5 successive scans in lateral and in the depth direction. Also, it was these microcracks that merged with the other microcracks resulted in micro-elevation as seen on the cortical bone surface.

**Table 1:** Table showing statistical analysis of average microcrack thickness/width, along with number of microcracks and micro-elevation found in each group analyzed in OCT images. Microcrack thickness and number of microcracks were measured at a depth of 100 µm below the bone surface of all OCT images for every sample group.

Measured Parameters	Experimental Groups								P- value
	Drill Free Method at 45°		Drill Free Method at 90°		Drill Method at 45°		Drill Method at 90°		
	Mean	Standard deviation	Mean	Standard deviation	Mean	Standard deviation	Mean	Standard deviation	
Microcrack Thickness^†^ (µm)	37.55	5.71	35.80	4.12	36.35	6.58	33.55	6.56	0.278
Number of Microcracks^‡‡^	5.40	1.14	6.95	1.19	3.75	0.91	4.50	1.32	<0.001**
Micro-elevation^‡^ (µm)	572.80	46.75	nil	Nil	489.35	60.17	nil	Nil	<0.001**

**p < 0.05.

^†^Analysis of Variance (ANOVA) was performed.

^‡‡^Kruskal Wallis ANOVA with post-hoc Conover test was performed.

^‡^Mann-Whitney U test was performed.

**Figure 5:**
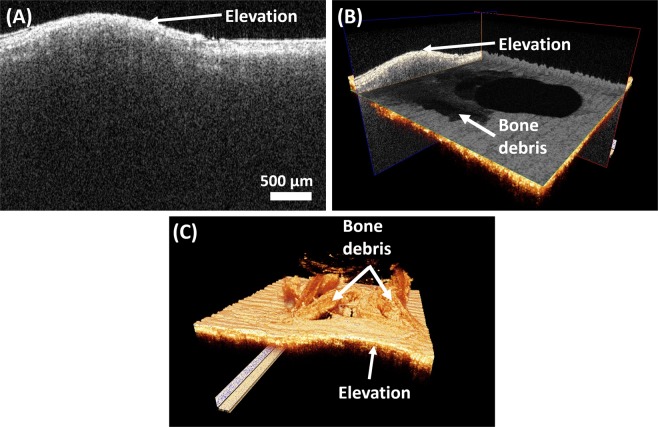
OCT images showing micro-elevation of the cortical bone surface following placement of microimplants at 45° angulation. (**A**) is a cross-sectional 2D OCT image, (**B**) is a 3D volumetric OCT image with orthogonal section planes, and (**C**) is a 3D volumetric OCT image showing micro-elevation of the cortical bone surface.

Figure 5 shows the bone micro-elevation due to a microimplant insertion at 45° angulation to the bone surface. We can appreciate the micro-elevation in figures (A) and (B) where the internal structures and layers of the bone are pushed to a level where individual structural layers of the bone are difficult to visualize. Also, the 3D volumetric image shows the accumulation of microdamage and the resultant fracturing of the cortical bone surface integrity following microimplant insertion. OCT images provide us with a detailed picture of the cortical bone structural integrity failure following microimplant insertion at a high resolution.

**Figure 6:**
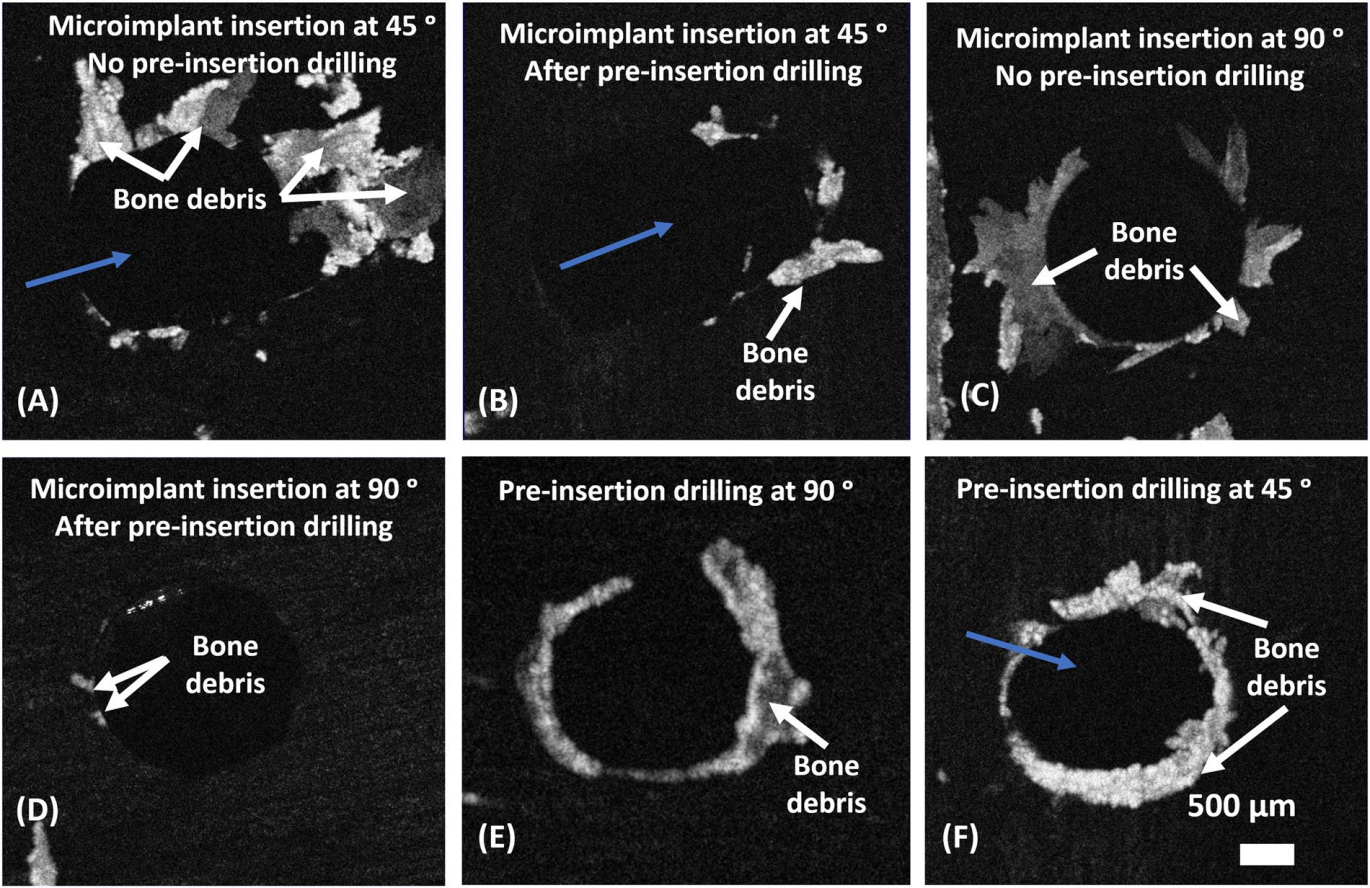
OCT *en face* images showing bone debris occurrence at the cortical bone surface, caused by microimplant insertion at 45° and 90°, with and without pre-insertion drilling performed. (**A**,**B**) Are respective *en face* images after microimplant placed at 45° in both the placement methods. (**C**,**D**) Are respective *en face* images after microimplant placed at 90° in both the placement methods. (**E**,**F**) Are *en face* images taken right after pre-insertion drilling at 90° and 45°. Blue arrows indicate direction of microimplant insertion.

To assess the bone debris formation upon microimplant insertion, we took *en face* images of the superior surface of bone samples of all four groups. Figure 6 shows a representative *en face* images in each group. (A) and (B) are the *en face* images which were taken from the 3D OCT image after drill and no-drill microimplant placement at 45°. Similarly, (C) and (D) are respective *en face* images seen at the top surface of the bone in a 3D volumetric image after drill and no-drill microimplant placement at 90°. It can be seen that microimplant placement after the drill method of insertion caused reduced bone debris when compared to no-drill microimplants placement method. Furthermore, the formation of bone debris is significantly larger, depending on the direction of microimplant placement. The blue arrows in Fig. 6, indicates the direction at which the micro implants were inserted on the cortical bone surface. (E) and (F) are the *en face* images taken right after pre-insertion drilling at 90° and 45° respectively. These OCT images were acquired before microimplant insertion. It is of significance to note that the process of drill method of insertion, led to the formation of bone debris, even though the amount of bone debris formation is comparatively less than the debris created by the no-drill microimplant insertion.

### Micro-CT imaging for bone damage evaluation upon microimplant placement

**Figure 7:**
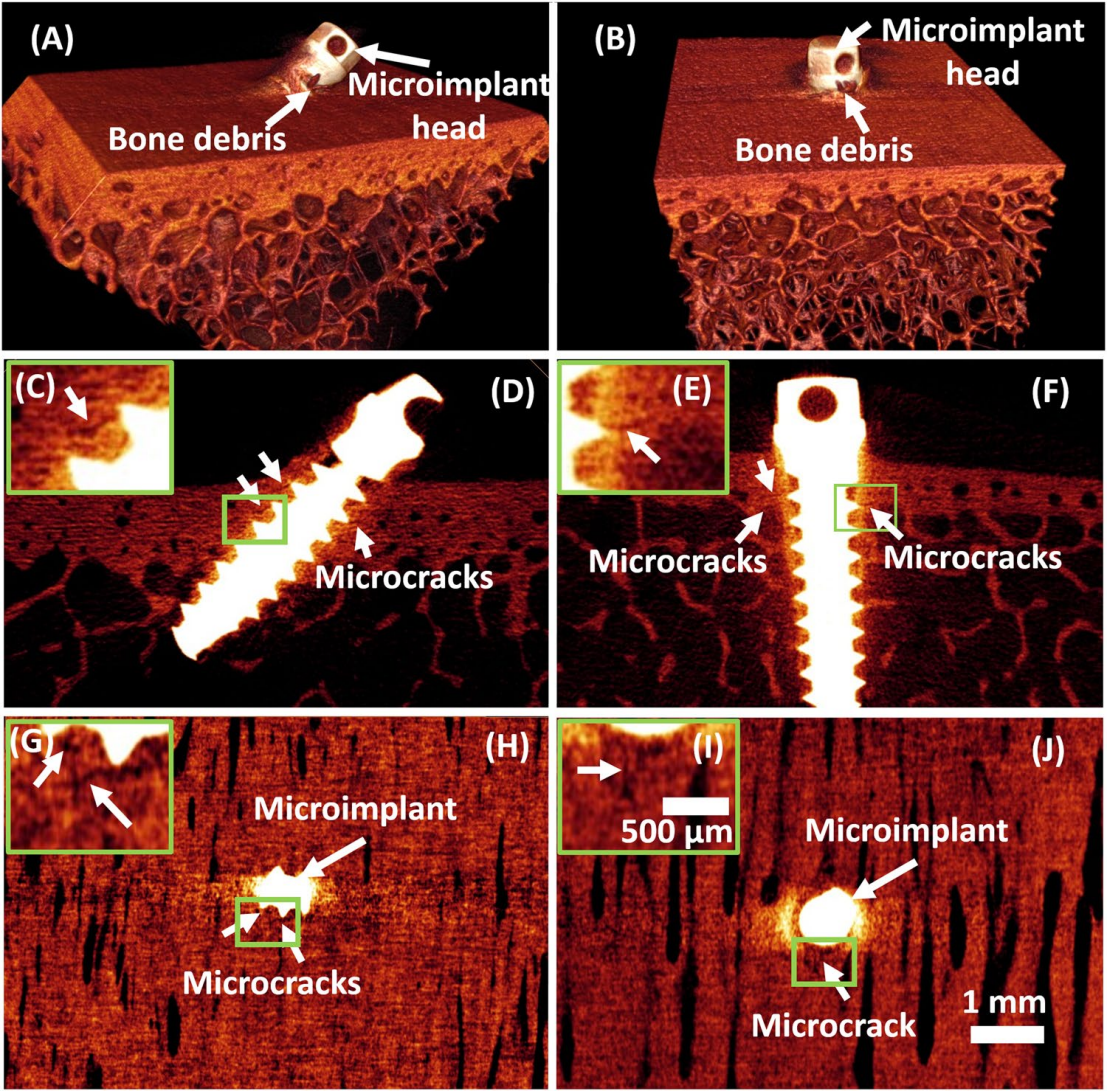
Representative 2D and 3D Micro-CT images of a micro-implant inserted to the cortical bone surface at 90 and 45° angulation. (**A**,**B**) 3D micro-CT images of microimplant mounted at 90° and 45° onto the bone surface. (**D**,**F**) Are 2D Micro-CT Images showing the cross-section of the sample along with the micro-implant at 90° and 45°. (**H**–**J**) *En face* images at 100 μm depth. Inset images (**C**,**E**,**G**,**I**) are magnified images shown in green box region shown in respective cross-section and *en face* images. Both the cortical and trabecular bone are visible in 2D and 3D micro-CT images. White arrows indicate the location of microcracks around the micro-implant.

To confirm our results of OCT imaging, we imaged the samples using micro-CT. Figure 7 represents the MICRO-CT images of the cortical bone after insertion of the microimplant. (A), (D) and (H) are 3D, 2D and *en face* images of the micro-implant inserted at an angle of 45° onto the cortical bone surface and (B), (F) and (J) are 3D, 2D and *en face* images of the micro-implant inserted at an angle of 90° onto the cortical bone surface. In the cross-section micro-CT images (D) and (F) the microcrack induced due to microimplant mounting is highlighted with white arrows, also the inset images are the magnified regions which are highlighted with green box in the respective Cross-sectional images. Similarly, the *en face* images at 100 μm below the bone surface for 90° and 45° microimplant inserted samples are shown in (G), (H), (I), and (J). Common scale bar for cross-sectional and *en face* images is given along with image (J) and a common scale bar for the magnified inset images is given along with (I).

## Discussion

The process of microimplant insertion into cortical bone leads to the generation of stresses in the cortical bone. Accumulation of stress in the cortical bone leads to formation of microdamage of the cortical bone as a method to relieve the stresses accumulating around the microimplant. The occurring microdamage can present itself as distinct or diffuse microcracks, as micro elevation of the cortical bone relative to microimplant placement and by formation of bone debris due to the screwing motion of the microimplant as it drills through the bone structure. To attain a more extensive picture of the accumulation of the stress, we ran a simulation of microimplant placement using the FEA method in two different angulations. As per the result of the simulation, we could concur that majority of the von misses stresses that were generated were at the subsurface level of the cortical bone in both the angulations and at the obtuse angle created between the microimplant and the bone surface. With this information in hand we then proceeded to the experiment phase of the research protocol.

As we could understand from the previous literature of OCT systems, we needed to remove any bias pertaining to the reflectivity of light, as it bounces of the metal surface of the microimplant and projects as a microcrack, leading us to a false positive result. Thus, we initially used a pilot drill to create holes on the bone surface at two different angulations and determined the presence of microdamage formation by scanning the pilot drill hole with the OCT system. The results of which are shown in Fig. 2.

After the scanning process, the image generated were transferred onto a volumetric software and processed. The image is projected into a series of images, each making up an individual slice of the compete image. For the purposes of removing any bias, to avoid false positive results and for facile reproduction of the results, we defined the microdamage to be; (a) microcrack, i.e., seen as a clear and distinct discontinuation of the cortical bone emanating from and around the microimplant surface. To be classified a microcrack, the actual crack needed to be present in 5 or more consecutive OCT images lateral and in the depth direction. (b) micro-elevation and (c) bone debris formation. Furthermore, we confirmed our findings with the use of micro-computed tomography, the scanning modality that is considered to be a gold standard in hard tissue imaging to determine the presence of microcracks in the cortical bone around the microimplant surface.

From the data presented in the study, we can derive OCT to be an effective tool to be used as a non-invasive imaging modality to detect and asses cortical bone microdamage occurring around the microimplant bone interface and the obtained images can be processed and analyzed to study the entities in a 3-dimensional manner.

Freshly extracted bovine bone samples were initially scanned to set a base line for the study. Following which, pre-microimplant holes were drilled and the samples were scanned. The samples were divided into four groups of 20 microimplants each and the microimplants were inserted onto the bone samples as prescribed by the manufacturer. After loading of the microimplants into the bone samples, individual samples were scanned using the OCT systems. With the use of our OCT system, we could achieve a depth of 250 μm into the cortical bone and this depth coincides with the high stress patterns previously shown in our FEA model. Even though, the penetration of the OCT system is not sufficient to image the micro-implant through its entirety, it provides images of adequate penetration depth and high resolution of the surface of the cortical bone.

As shown in Fig. 3, the areas of microdamage around the microimplant-bone interface are clearly visible in the OCT images. Microdamage, as defined, is the combination of microcracks, micro elevation and bone debris formation, collectively effecting the structural integrity of the cortical bone around the microimplant and in OCT images is seen as areas of different intensity and elevations on different planes as compared to the surrounding normal bone surface. These affected areas are seen around the implant surface and propagate to an area of up to 1 mm.

Depth intensity profile analysis was performed using Matlab coding, to confirm and evaluate the presence of micro cracks around the bone-implant interface. The sudden drop in the intensity corresponds to the discontinuity in the bone structure around the implant, validating the visualization of micro cracks present in the 3D model. This feature of OCT sets it apart from other commonly used imaging modality, as quantitative analysis pertaining to the nature of the micro crack can be obtained. In the following study, Fig. 4 represents the depth intensity profile analysis of the obtained OCT images. Following the depth intensity profile analysis, the results of the quantitative analysis of the occurring microdamage is tabulated in Table 1 as shown.

Area of microdamage cover is noted to be more when microimplants are placed in the no-drilling method and visibly lower when a pre-drill hole is made prior to the microimplant insertion. This could be due to the additional torque that needs to be generated whilst placement of the microimplant in the no-drill method. Additionally, when the microimplant is placed at an angle of 45° in both the insertion methods, the occurring microdamage is increased, owing to the fact that when we place the microimplant at an angle, there is more engagement of the cortical bone with the microimplant leading to increased insertion torque and subsequently increased microdamage^23^.

Primary stability of the microimplant is a determining factor for the success of the microimplant. Quality of the bone structure is a vital aspect of primary stability, and the occurring bone microdamage can directly affect the stability of the microimplant. As observed in Figs 3 and 4, microcracks can be visualized as minute discontinuity of the cortical bone around the implant. Numerous microcracks are present, but only a few large microcracks are seen navigating around the mini-screw implant. Primary stability of the implant depends on the inter-locking mechanism between the mini-screw implant the bone surrounding the implant. During the process of insertion, the torque generated can lead to the formation and propagation of microcracks through the bone structure^24,25^. Microcracks can cause a significance effect to the primary stability of the implant. Large microcracks can develop into areas of weak bone structure and compromise the balance at the bone-implant interface, leading to failure of microimplants^26^. In our study, we noticed the presence of microcracks all around the implant, especially correlating with the direction of placement of the microimplants and the method of microimplant placement. An increase in the number of cracks was noticed corresponding to the angulation of mini-screw implant placement and the mode of microimplant placement. When the implant was placed at an angle of 45°, an increase in number of microcracks was seen, as when compare with 90° angle placement. This could be due to the fact that, when placed at an angle of 45°, the implant transverses more on the cortical bone as compared to 90° angulation^27^. Operator dexterity and precision during application of force whilst implant placement can be a reason for the occurrence of microcracks on the bone structure. High resolution and high contrast images generated by OCT, benefits the visualization of the microcracks around the bone-implant interface. Furthermore, utilizing the 3D scans, a comprehensive analysis of the microcracks direction and means of propagation can be attained using OCT technique.

Micro elevation is another important aspect effecting the primary stability of the microimplant. Angular placement of the microimplant, causes the microimplant to transverse more in the denser cortical bone. As when the threads of the microimplant engages the dense cortical bone, the bone material is pushed upwards as the microimplants transverses forward, leading to areas of micro elevation of the cortical bone, at the superior angle formed between the microimplant and bone surface. These areas as shown in Fig. 5, act as areas of demineralization around the microimplant and can affect the primary stability of the microimplant.

Accumulation of bone debris along the surface of the bone adjacent to the microimplants is of note (Fig. 6). Presence of the bone debris, its shape and quantity are easily evaluated though OCT imaging. In *en face* images rendered from 3D volumetric OCT images (Fig. 6(B–F)), bone debris can be observed. Bone debris, in the images is seen as large masses of opaque intensities accumulating around the implant head. The presence of these bone debris is due to the non-drilling nature of the microimplants and do not pose a threat to visualize the microcracks on the scanned images. Although, we would advise cautious removal of these debris for better evaluation of the surface underneath the debris. Depth penetration ability of OCT is of added value in relation to the visualization of micro-damage and microcracks on the bone structures, as using the 3D modes, we can navigate through sections underneath the debris to locate and analyze these structures. However, from a clinical point of view, the accumulation of the bony debris adjacent to the microimplant and within the screw threads can lead to an increase of stress within the cortical bone leading to disruptions on the implant-bone interface^26^.

We confirmed the results of our study, via the means of micro-computed tomography. Micro computed tomography, of late is considered the gold standard for assessing bone morphology and microstructures^28^. As seen in OCT images, the areas of microdamage is distinctly and more accurately seen in MICRO-CT images, but, the radiation exposure associated with micro-CT is very high. The ionizing effects of the radiation can have an effect on samples. Secondly, the micro-CT imaging technique is relying on the contrast, brightness and signal to noise ration. These parameters are sensitive and can lead to alterations in the image quality. Some imaging artefacts are peculiar to CT, such as ring artefacts and beam hardening and can affect the overall quality of the image.

From a clinical point of view, the prevalence of microdamage, plays a significant role in the primary stability of the microimplant. As seen in our study, placement of non-drilling microimplants at an angle of 45°, caused the maximum damage to the cortical bone. In these cases, the failure rate of microimplant will be increased. Alternatively, we notice minimal microdamage to the cortical bone when the microimplant is placed more perpendicular to the bone surface and when a pre-drill hole is placed before the insertion of the microimplant. We can concur that placement of microimplants more perpendicular to the bone surface and by utilization of a pre-drill hole is a better modality for the success of microimplants.

The most important feature of primary stability of microimplants is represented by the intimate contact between the implant and the bone. To investigate such aspects, it is essential to have a noninvasive method to predict the stability of the inserted implants. To this goal, the OCT method could operate non- invasively and allow the orthodontists to evaluate the success and to predict the efficiency of the orthodontic microimplant treatment. The usual investigative methods known such as histological studies and computed tomography are invasive and they exhibit inferior resolution and contrast as compared to OCT imaging. This added information, can contribute to a better prognosis of orthodontic treatment.

## Conclusion

It is evident from our study that, OCT can be used as a valid and reliable imaging modality to study the microcracks formation on the surface of the cortical bone and its propagation through the bone. The associated microdamage to the cortical bone is adequately visible and quantitative analysis of the microcracks as it propagates through the cortical bone can be performed using the A-scan analysis of the OCT images. In our study, we see that there is an increase in bone microdamage following placement of microimplants by the no drill method and an increase in bone microdamage is seen following placement of microimplants at an angle to the cortical bone surface. Hence, a better stability of microimplant can be derived with a microimplant that will be inserted perpendicular to the cortical bone surface and utilizing a pre-drill before insertion. Furthermore, with technical advancement, real-time imaging of the bone surface can be achieved, especially since no harm is done to the patient’s due to OCTs non-invasive quality. Further studies need to be made utilizing the full spectrum of options available with OCT imaging.

## Methods

### Finite Element Analysis

A finite element analysis of the microimplant-bone structure was carried out using ANSYS system (ANSYS R15.0, Ansys Inc., Houston, PA, USA). The microimplant was designed with a shaft length of 7 mm, with a lower screw diameter of 1.2 mm, and an upper diameter of 1.3 mm. pre-saved micro-CT images were saved in Digital Imaging and Communications in Medicine (DICOM) format. Following which the three-dimensional modelling of the microimplant using micro-CT slices was performed using a CT modeler. This enabled for a rapid processing 3D DICOM data with an automatic segmentation tool, from which the 3D finite element model of the microimplant was constructed. The bone region was shaped as a rectangle. The cortical bone and the microimplant were modeled as isotropic, homogeneous and linear elastic material. The modeled microimplant were inserted onto the cortical bone in two different angles, 90° and 45°. The microimplant screws were designed with 10878 nodes and 48917 elements, and for the bone structure it varied depending on the insertion. For 45° insertion we used 5292 nodes with 16391 elements, and for 45° apex it was 4566 nodes with 15215 elements. For 90° insertion it was 4574 nodes with 14879 elements, and 90° full apex it was 4554 nodes and 14790 elements. And the analysis determined the area of stress on the bone tissue around the implant once the microimplant is completely inserted into the cortical bone. Von Mises equivalent stress values were used to assess critical areas of the microimplant-bone structure as shown in Fig. 1.

### Specimen Preparation

**Figure 8:**
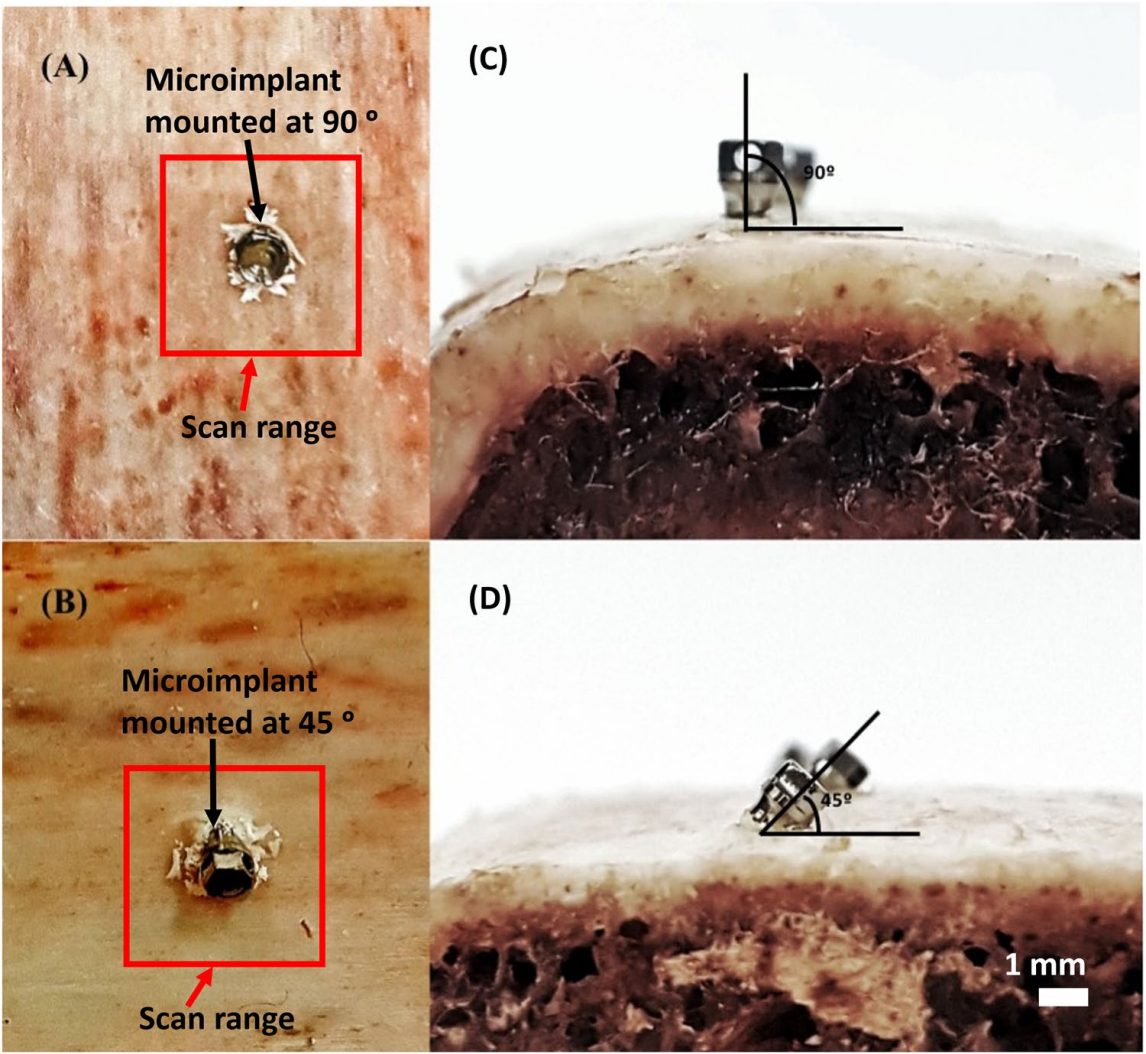
Representative photographs of microimplant insertion onto the cortical bone surface. (**A**,**C**) Are photographs of microimplant inserted at an angle of 90° to the bone surface. Likewise, (**B**,**D**) are photographs of microimplant inserted at an angle of 45° to the bone surface.

Bredbenner *et al*.^29^ have shown that freshly harvested bovine rib has material properties similar to human bone, with clear definition of cortical and cancellous bone and is a material of choice for studies focusing on maxillofacial implantation. In order to make experiment setup similar to *in-vivo*, we used bovine rib bone segments which were freshly harvested and stored in a cool dry place for less than 10 hours prior to the experiment. The bone specimen segments were then cut into workable pieces of same dimensions to serve as microimplant placement sites, shown in Fig. 8. There is a possibility that the increased bone density may produce more bone damage with no drill method and angular placement. This was also one of the reasons for why we opted to use sample specimens of same bone density for our experiment. The specimen segments were derived of its periosteum and stored in saline until use. (A) and (C) are photographs of a microimplant inserted at an angle of 90°, similarly, (B) and (D) are photographs of a microimplant inserted at an angle of 45° to the cortical bone surface.

### Microimplant Armamentarium

A total of 80 conical-shaped titanium microimplants of the AbsoAnchor System (Dentos, Daegu, Korea) with identical design (NH 1312-07) was selected for this study. Among the various available microimplants used for orthodontic clinical practices, the microimplant used in this study is one among the commonly used in clinical practices. Each of the microimplant was 7 mm in length and had a diameter of 1.3 mm. The microimplants used were of two different types, namely, self-drilling microimplants and self-tapping microimplants. The microimplants were grouped into four groups, with 20 implants in each group. The microimplants were inserted utilizing two different methods of insertion, i.e., (a) drilling method and (b) no-drill microimplant placement method. For the pilot drill, Dentos pilot drill was used (PD-31-0.9) on a slow hand piece at a speed of 600–700 rpm. With each method the microimplants were inserted at an angle of 90 and 45° on to the bone segments using the tools provided by the company, as shown in Fig. 2, respectfully. Following microimplant insertion, the bone segment along with the microimplant was mounted on an acrylic segment to stabilize it from untoward movement during scanning.

### Optical Coherence Tomography

#### OCT specification

For the proposed experimental study, a commercially available swept source optical coherence tomography system (OCS1310V1; Thorlabs, Newton, NJ) was utilized. The OCT system was used to obtain cross-sectional (2D) and volumetric (3D) images. The system is connected to a preconfigured personal computer and the images are obtained with a scanner probe. The commercial system had a center wavelength of 1300 nm with a spectral bandwidth of >97 nm, and the axial resolution of the system can be attained <16 µm and a transverse resolution of 25 µm. The scan range was set to 4 mm to obtain one 2D image. And to acquire one 3D volumetric image a scan area of 4 × 4 mm was set. The OCT system produces 3D images by combining successive 2D scans in lateral direction within the area of interest, thereby allowing to perform volumetric and depth analysis of the bone surface.

#### Depth intensity profile analysis

Depth intensity profile analysis was carried out on 2D images to analyze the cracks, bone debris, and bone elevation caused due to microimplant placement in bone samples. For this the obtained 2D images were processed with the help of a MATLAB program (MATLAB 2014a, The MathWorks, Natick, 2014). The developed code first applies a median filter of 2 × 2 to the selected 2D OCT image, followed by a sequential analyses of the OCT 2D images are performed. Upon determining the point of intensity, profile analysis was performed. The obtained corresponding intensity profiles were normalized and plotted. This intensity plotting of OCT images were performed in both the lateral and horizontal direction as per the requisite. Since the OCT images are representations of layers/ structures which differ in intensity with accordance to the changes in the refractive index of sample structures, the intensity or the absence of it, represents either the sample structure is less than the system resolution or refractive index of structure is varied accordingly. In our experiment, the depth intensity profile plots which were taken in the region of microcracks can be seen as abrupt/ sudden fall in the intensity of the plots, which progresses to few millimeters depending on the crack width. Similarly, we correlated the cracks, bone debris, implant surface, and other structural properties of the bone samples resulting from microimplant placement.

### Micro-Computed Tomography Imaging

The three-dimensional microstructure of the bone sample was estimated by micro-computed tomography (Skyscan 1076, Skyscan, Kontich, Belgium) using a 15 μm low-contrast resolution, which was powered by a sealed x-ray source of 20–100 kV with a spot size of less than 5 mm (4 W). The camera pixel size of micro-CT was 12.25 μm and source voltage was 100 kV. Exposure was for 680 ms and the rotation step was 0.600 degrees. The scanning motion was step and shoot. The single scan duration was 24.26 minutes and radiation safety were less than 1 μSv/h at any point on the instrument surface during the scan. Total scanning volume was 17 μm/ single scan lengths. Image processing was performed using volume rendering software on a personal computer.

### Ethics

We deem our study does not require any ethical clearance as it does not involve live human or live animal participation or personal data, consulting the relevant faculty, department, school and university policies and personnel and since minimal risk of any sort was identified.
